# Nesting and Foraging Preferences of Four Sympatric Species of Cavity-Nesting Leafcutting Bees (Hymenoptera: Megachilidae)

**DOI:** 10.3390/insects16080831

**Published:** 2025-08-11

**Authors:** Qianlei Dai, Junjie Hu, Xuan Liu, Jia Wan, Jiabao Wei, Dongshuo Yang, Chunling He

**Affiliations:** 1 College of Horticulture and Plant Protection, Henan University of Science and Technology, Luoyang 471000, China; d1713821911@163.com (Q.D.); hujunjie617@163.com (J.H.); 15036679391@163.com (X.L.); 18438669750@163.com (J.W.); 13592082539@163.com (J.W.); 2 College of Life Sciences, Luoyang Normal University, Luoyang 471934, China; yangdongshuo@126.com; 3 The Observation and Research Field Station of Taihang Mountain Forest Ecosystems of Henan Province, Xinxiang 453007, China

**Keywords:** solitary bees, trap nests, nesting substrate, Megachilidae, pollen analysis

## Abstract

**Simple Summary:**

Using a total of 1451 nesting traps, we documented the seasonality, nest architecture, and forage plants of the following four sympatric species of Megachilidae from the Taihang Mountain Nature Reserve in Henan, China: *Megachile spissula* Cockerell, *Megachile sculpturalis* Smith, mason bees *Osmia taurus* Smith, and *Anthidium septemspinosum* Lepeletier. The nesting activity period in trap nests occurs primarily from late spring through summer, with temporal segregation in peak activity among species. Each species exhibits distinctive characteristics in the types of materials used for constructing brood cells and nest plugs. Female bees were recorded collecting pollen on 48 plant species from 24 families. Among these, 14 plant species served as shared pollen sources, representing 29.17% of the total utilized plant taxa. These results offer important references for conservation efforts.

**Abstract:**

Megachilidae are crucial pollinators of cultivated and wild vegetation, playing a vital role in ecosystem pollination services, however, there is still a lack of information regarding the ecology and behavior of these species. This study aims to analyze the nesting ecology strategies of four sympatric species of leafcutting bees and their interactions with pollen source plants. Data were collected from April to October from 2019 to 2022 in the Jiyuan section of the Taihang Mountain National Nature Reserve (approximately 35°10′–35°25′ N, 111°55′–112°10′ E) using trap nest methods. Through the dissection of nesting tubes, their structural characteristics were revealed, and the pollen sources collected by the bees were identified. Our results showed that nesting activity of leafcutting bees lasted from May to October, with a preference for nesting tubes of 6 to 10 mm in diameter and 131 to 170 mm in length. We documented 48 plant species used as foraging sources, belonging to 17 orders, 24 families, and 33 genera, with the Fagaceae family (9 species) being predominant. The results indicate that the distinctive traits of these species—such as the asynchronous nesting periods, the types of nesting materials, the dimensions of cavities, and differential utilization of floral resources—likely play a critical role in niche differentiation among sympatric species, thereby ensuring the maintenance and persistence of Megachilidae populations in this region.

## 1. Introduction

Pollinating insects play a crucial role in global ecosystems, providing essential services that are vital for the diversity of wild plants, human dietary structures, agricultural production, and even economic development [1,2]. Among these, bees are the primary pollinators of flowering plants [3,4]. Of the approximately 20,000 species of bees, the vast majority are wild species, accounting for about 85% of the total bee population [5,6]. The global decline in pollinator populations, particularly bees, poses a significant threat to the productivity of major crops, vegetables, and fruits [7,8]. Factors contributing to the decrease in bee populations include climate change, pesticide overuse, habitat degradation, predators, and parasites [9,10,11]. Providing suitable nesting habitats and foraging resources may aid in mitigating their decline and sustaining their populations [12,13].

The family Megachilidae is widely distributed globally, with approximately 4000 species worldwide. China has recorded 305 species and subspecies [14,15,16,17]. Bees in the Megachilidae family possess long tongues and are important pollinators of long-corolla plants. Their abdominal ventral surface features a pollen brush (scopa) for pollen collection (in contrast to many other bees that rely on their hind legs). With these outstanding morphological characteristics and adaptive interactions, they exhibit exceptional pollination efficiency on crops, trees, forage grasses, and other plants, making them more effective pollinators in the pollination processes of most plants [18,19,20]. Notably, *Megachile rotundata* is widely used for the pollination of forage crops such as alfalfa and clover in Europe and America [21,22]; *Osmia lignaria propinqua* is employed in the United States for the pollination of fruit trees like almonds and apples [23]; *Eumegachile pugnata* is utilized for sunflower pollination [24]; and *Osmia cornifrons* is used in Japan for the pollination of fruit trees [25].

Most species in the Megachilidae family are solitary, nesting primarily in soil and preferring to utilize existing cavities such as pithy stems, galls, and decaying wood [18,26]. Based on their morphology and the materials they use to arrange larval brood cells, Nesting leafcutter bees are broadly categorized into four groups: (1) mason bees, which predominantly use mud and chewed plant materials; (2) resin bees, primarily utilizing plant resins; (3) true leafcutter bees, which mainly cut and use fragments of living leaves; and (4) wool carder bees, mainly combing and using soft materials such as plant fuzz and fine fibers [27]. Different species may collect one or more types of natural materials to construct their brood cells; some even use glandular secretions to line their cells [28]. Each nest consists of a series of linear brood chambers, within which the female bee collects and stores a pollen food mixture (a blend of nectar and pollen).

The nest method is currently the optimal approach for monitoring the nesting and foraging behaviors of cavity-nesting bees [29,30]. Different species select distinct nest tube structures; statistical data reveal that the majority of bees prefer nest tube lengths ranging from 1.4 cm to 28 cm, with an average length of 11.3 cm. The diameters of these tubes vary from 2 mm to 25 mm, with an average diameter of 7.2 mm [31,32,33]. For instance, Karsten (2015) found that *Osmia bicornis* prefers nest tubes with an inner diameter of 8–10 mm and a length of 150 mm [34]. Ivanov (2013) noted that *Osmia dimidiata* occupies cavities between 8 cm and 28 cm long, favoring cavities that are 15–20 cm in length [35]. Dos Santos et al. (2020) reported that *Megachile zaptlana* prefers cavities with diameters ranging from 5 mm to 10 mm, with a preference for 6 mm [26]. Payne (2011) indicated that *Anthidium manicatum* utilizes cavities with inner diameters of 9.5–15.6 mm and lengths between 79 mm and 222 mm [36]. Vitale et al. (2017) discovered that, among four species of *Anthidium*, *A. andinum*, *A. decaspilum*, and *A. vigintipunctatum* favor cavities with an aperture of 8 mm, while *A. rubripes* prefers cavities with a diameter of 5 mm [37]. With the current global decline of bees worldwide, a few studies have revealed that megachilids and other cavity-nesting bees are threatened by several factors such as climate change and habitat loss [38,39]. Therefore, the current study is addressing the conservation of four megachilid species in the South Taihang Mountain region, where artificial nest tube techniques were employed from 2019 to 2022 to investigate the nesting and foraging preferences of four sympatric species of leafcutter bees.

## 2. Materials and Methods

### 2.1. Study Area

**Figure 1:**
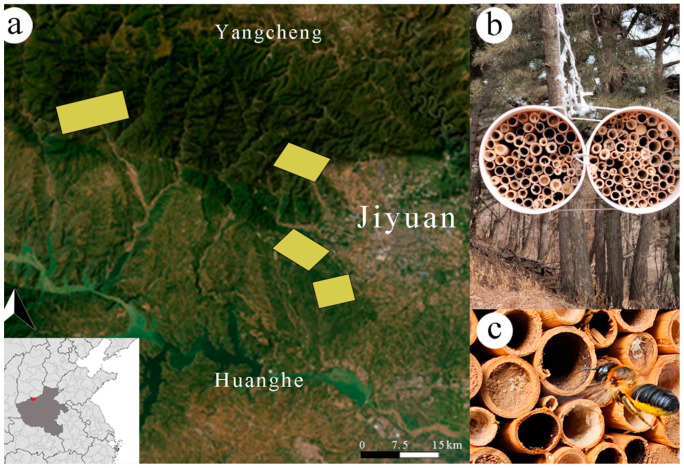
Figure illustrating the study area and nesting tubes: (**a**) Research site within the Taihang Mountain National Nature Reserve (Jiyuan section, Henan Province); sampling points are highlighted in yellow. (**b**) Schematic diagram of artificial nesting tubes. (**c**) Leafcutting bee nesting tubes.

The study was conducted from March 2019 to October 2022 in four forest areas of the Taihang Mountain National Nature Reserve (Jiyuan section, Henan, China) (Figure 1a). Jiyuan is located in the northwest of Henan Province, with geographical coordinates ranging from 34°54′ N to 35°16′ N and 112°02′ E to 112°52′ E, at elevations between 150 m and 1955 m, covering a total area of 1931.5 km^2^. The climate is a warm temperate continental monsoon climate, with an average annual temperature of 14.3 °C. Precipitation is primarily concentrated between June and September, with an average annual rainfall of 641.7 mm. The average frost-free period is 213.2 days, with an average annual sunlight duration of 2044.2 h and an average annual sunshine rate of 46%.

### 2.2. Bee Capture

The nesting boxes consist of two white PVC pipes, each 25 cm in length and with an inner diameter of 10 cm, secured in place with iron wire at the field sampling locations. The pipes are filled with reed tubes measuring approximately 20 mm in length and with inner diameters ranging from 3 to 12 mm. The nesting boxes are suspended approximately 1.5 m above the ground (Figure 1b), with any surrounding shrubs and branches that could obstruct access cleared away. They are installed in open, sunlit areas, with the nest openings oriented east–west [40]. From May to October in the years 2019 to 2022, the occupied nest tubes were regularly collected once a month, and empty tubes were replenished. All collected tubes were sorted according to their nesting tube numbers and taken back to the laboratory for rearing and observation.

### 2.3. Nest Structure

In the laboratory, the collected nest tubes were sequentially numbered according to the nest box identifier and dissection order. A scalpel was used to incise each nest tube at one-third of its diameter, and measurements of the tubes (diameter and length) were taken using an electronic caliper and a ruler. Additionally, the number of brood chambers and empty chambers within each tube was recorded. Finally, the dissected nest tubes were placed into dry glass test tubes measuring 250 mm in length and 25 mm in diameter, which were sealed at the openings with medical-grade absorbent cotton. The tubes were maintained at room temperature for subsequent observation and recording.

### 2.4. Measurement of Morphological Characteristics

Morphological characteristics of nesting bees were captured using a Zeiss 3D microscope (Smartzoom 5, ZEISS, Oberkochen, Germany) and a macro camera. A caliper was employed to measure the morphological indices of the nesting bees. An electronic caliper was used to determine the body length, thorax width, and other morphological metrics for 10 specimens each of male and female bees, with measurements expressed in millimeters (mm).

### 2.5. Identification of the Pollen Spectrum Within Bee Nests

Equal quantities of pollen blocks from different nesting chambers of the same bee species were collected and mixed to form a single sample. Each sample was then added to liquid nitrogen, thoroughly homogenized during grinding, and 100 mg was used for DNA extraction.

PCR amplification and sequencing: The ITS2 region of the nuclear genome of the pollen was amplified using the primers ITSS2F: 5′-ATGCGATACTTGGTGTGAAT-3′ and ITS4R: 5′-TCCTCCGCTTATTGATATGC-3′. The PCR reaction mixture consisted of 20 μL, comprising: 2 μL of DNA template (5 ng/μL), 4 μL of FastPfu Buffer (5×), 2.5 μL of dNTPs (2.5 mmol/L), 0.8 μL of each primer (5 μmol/L), 0.4 μL of FastPfu Polymerase, with ddH2O added to a final volume of 20 μL. The PCR conditions were as follows: initial denaturation at 95 °C for 5 min; denaturation at 95 °C for 30 s, annealing at 55 °C for 30 s, and extension at 72 °C for 45 s, repeated for a total of 29 cycles; final extension at 72 °C for 10 min; and storage at 12 °C. The PCR products were analyzed using 2% agarose gel electrophoresis and subsequently purified using the AxyPrep DNA Gel Extraction Kit (Axygen Biosciences, Union City, CA, USA). The purified products were sent to Shanghai Lingwen Biotechnology Co., Ltd. (Shanghai, China) for paired-end sequencing (2 × 250 bp) on the Illumina MiSeq platform.

### 2.6. Data Analysis

All statistics were performed in R Studio (4.4.2). Environmental variable data (e.g., temperature, precipitation, wind speed) were obtained from http://eia-data.com/ (accessed on 1 September 2024). The number of individuals captured per month and year was recorded, and Pearson’s correlation coefficient was employed to assess the relationship between the total abundance of bees in each sampling year and the environmental parameters. Differences in nesting diameter, length, and number of nesting chambers among different species were tested using the Kruskal–Wallis test. To evaluate the effects of tube length on nesting diameter and the number of nesting chambers, generalized linear regression analysis was conducted using ordinary least squares (OLS), with nesting diameter and number of nesting chambers as dependent variables and tube length as the independent variable.

## 3. Results

### 3.1. The Number of Leafcutting Bees

**Figure 2:**
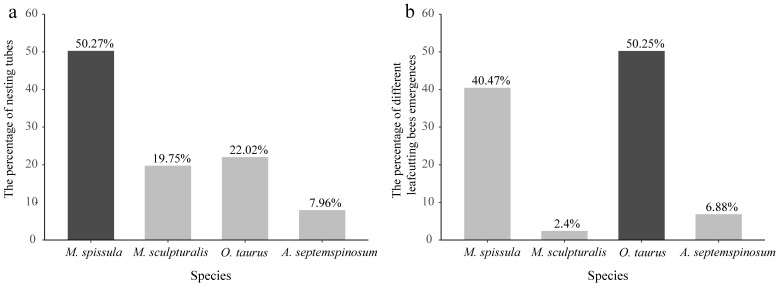
The number of nesting tubes collected and the number of adult emergences in four species of leafcutting bees collected between 2019 and 2022. (**a**): Number of nesting tubes collected. (**b**): Number of leafcutting bee emergences. Dark color indicates the species with the highest quantity.

During the period from 2019 to 2022, four species successfully nested in the studying area: two species of leafcutting bees, *Megachile spissula* Cockerell, 1911 and *Megachile sculpturalis* Smith, 1853, one species of mason bee, *Osmia taurus* Smith, 1873, and one wool carder bee, *Anthidium septemspinosum* Lepeletier, 1841. In total, 1451 nesting tubes were collected, with a total of 5087 nesting bees. The numbers of nesting tubes and emerged bees varied among the four species, with *M. spissula* having the highest number of nesting tubes, accounting for 50.27% of the total (Figure 2a), while *O. taurus* had the highest number of emerged adults, representing 50.25% of the total (Figure 2b).

### 3.2. Nesting Activity Duration

**Figure 3:**
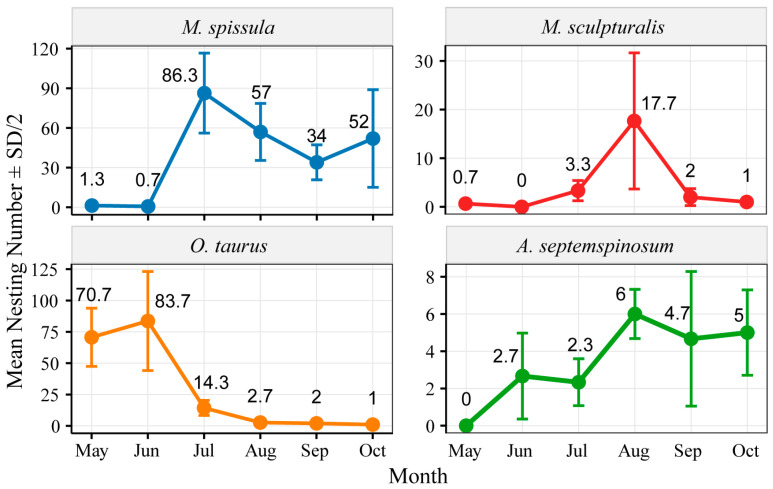
Monthly nesting activities of four leafcutting bee species from 2019 to 2021 (error bars represent half the standard deviation).

From 2019 to 2021, the nesting activities of four leafcutting bee species occurred from May to October, with distinct staggered peaks observed in the nesting activities of the four bee species. Among them, the nesting quantity of *M. spissula* exhibited a unimodal distribution, reaching its peak in July (approximately 86.3 individuals, accounting for 37.31% of the total observations). *M. sculpturalis* displayed a late peak-type pattern, initiating nesting in July, reaching its peak in August (17.7 individuals), and displaying a small amount of nesting in September. *O. taurus* showed an early peak-type nesting pattern, with June as the peak nesting period (around 83.7 individuals, representing 47.99% of the total count). *A. septemspinosum* had relatively fewer nesting activities (an average of 3.5 individuals per month) with no obvious peak between months (Figure 3).

### 3.3. Correlation Between Bee Abundance and Temperature, Rainfall, and Wind Speed

The correlation between bee abundance and environmental variables indicates that, over four years, temperature and rainfall are positively correlated with bee abundance, with temperature consistently showing significant correlation while rainfall correlation is not always significant. Temperature exhibited the highest correlation values over the four years, with the most significant values observed between 2020 and 2022, whereas rainfall showed the most significant correlation values between 2020 and 2021. Wind speed showed a slight positive correlation with bee abundance in 2020 and 2022, and a slight negative correlation in 2019 and 2021, with correlations not being significant over the four-year period (Table 1).

### 3.4. Nesting Material

**Figure 4:**
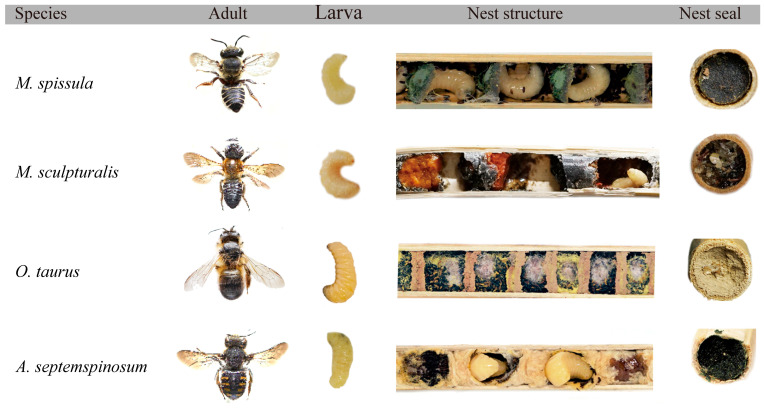
The morphological characteristics of larvae and adults, internal structure of nests, and sealing materials in four species of leafcutting bees. Each species exhibits distinct nest partition and sealing materials, as well as unique external appearance.

All four bee species utilize hollow nesting tubes for construction, but they differ in the materials used (Table 2). *M. spissula* utilizes circular leaves as partition materials, occasionally incorporating mixtures of brown soil; the thickness of the leaf closures is generally greater than that of the internal partition walls, and upon exposure to air, their color darkens to black and gradually hardens. *M. sculpturalis* utilizes a mixture of resin and soil for the partitions, while the closures are made from a combination of soil, pebbles, and resin. *O. taurus* employs soil as the material for nest partitions and closures, resulting in a tightly arranged nest with thicker closures. *A. septemspinosum* predominantly uses leaves and fluff for nesting, forming individual chambers wrapped in cotton-like material. When the female has laid all her eggs and there is still ample space remaining in the tube, she fills the leftover space up to the tube opening with cotton fluff. Finally, she chews leaves into small fragments and mixes them with secretions to apply a thin layer of closure at the entrance for reinforcement and camouflage (Figure 4).

### 3.5. Nest Inner Diameter and Length

**Figure 5:**
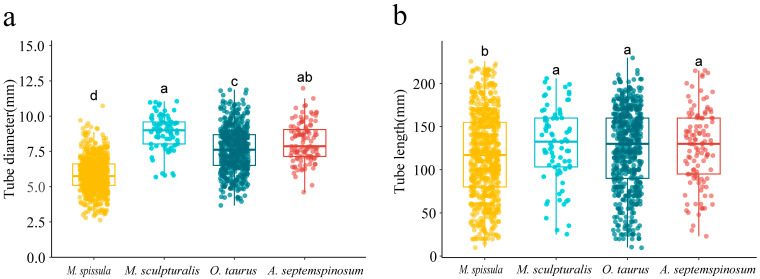
The nest tube diameter (**a**) and length (**b**) preferences of four species of leafcutting bees were examined. Different letters above the boxplots indicate significant differences in preferences between species (Kruskal–Wallis test, *p* < 0.05).

The nesting ecology of four leafcutting bee species shows that 90% of nest inner diameters range from 4–10 mm, with the highest proportion found for nest inner diameters of 6–10 mm at 69% (Table 3). Significant differences in nest inner diameters among the four bee species were observed (Kruskal–Wallis χ^2^ = 544.37, df = 3, *p* < 0.05, Figure 5a). The thorax width of female *M. spissula* is 2.85 ± 0.22 mm, preferring nest cavities of 6–8 mm. Similarly, female *O. taurus* and *A. septemspinosum* have thorax widths of 5.10 ± 0.66 mm and 4.35 ± 0.34 mm, respectively, both preferring nest cavities of 6–10 mm. Female *M. sculpturalis*, with a thorax width of 6.81 ± 0.49 mm, favors nesting in cavities of 8–10 mm.

Ninety percent of the nesting lengths of the four leafcutting bee species are from 30–195 mm, with the most common nest tube lengths falling within the range of 131–170 mm (30%) (Table 4). There are significant differences in nest tube lengths (Kruskal–Wallis χ^2^ = 11.962, df = 3, *p* < 0.05, Figure 5b), with *M. spissula* having significantly shorter nest tube lengths compared to the other three bee species. The body length of female *M. spissula* is 9.70 ± 0.73 mm, and they prefer nest tube lengths of 91–130 mm. The body lengths of female *O. taurus*, *A. septemspinosum*, and *M. sculpturalis* are 5.10 ± 0.66 mm, 13.29 ± 0.94 mm, and 23.02 ± 1.93 mm, respectively, with these three bee species showing a preference for nesting in nest tubes of 131–170 mm in length.

### 3.6. Brood Cell

Significant differences were observed in the total number of nest chambers and brood chambers among the four species of leafcutting bees (Kruskal–Wallis χ^2^ = 336.51, df = 23, *p* < 0.01; χ^2^ = 365.66, df = 22, Table 5). Osmia taurus exhibited the highest average total number of nest chambers (9.88) and brood chambers (8.93), significantly exceeding the other species (*p* < 0.01), with *M. sculpturalis* having the fewest chambers (Table 5). Specifically, the total numbers of nest chambers (5.53) and brood chambers (5.32) of *A. septemspinosum* were very close, suggesting minimal empty chambers during nest construction, while other species had approximately one empty chamber. The Pearson correlation coefficient analysis revealed a strong positive correlation (r = 0.95) between the total number of nest chambers and brood chambers, indicating that an increase in total nest chambers is associated with a higher number of brood chambers.

**Figure 6:**
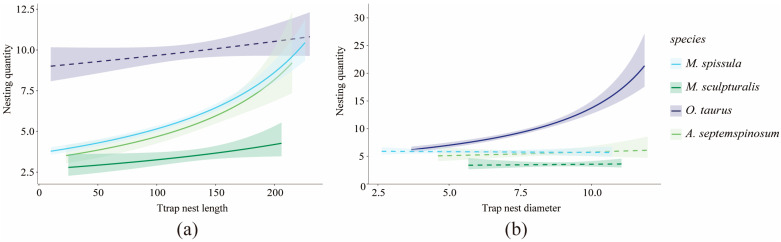
Generalized linear models of tube length and diameter in relation to nesting quantity in bees. (**a**): Trap nest length and nesting quantity; (**b**): Trap nest diameter and nesting quantity. The shaded area represents the 95% confidence interval. Solid lines indicate significant changes (*p* < 0.05), while dashed lines indicate non-significant changes (*p* > 0.05).

Analysis using generalized linear models revealed that the number of chambers for *O. taurus* significantly increases with the internal diameter of the nest (*p* < 0.05). In contrast, the chamber numbers for *M. spissula*, *A. septemspinosum*, and *M. sculpturalis* did not show significant changes in relation to the internal diameter of the nest (*p* > 0.05) (Figure 6a). For *M. spissula*, *A. septemspinosum*, and *M. sculpturalis*, the total number of chambers significantly increased with nest length *(p* < 0.05). However, for *O. taurus*, the variation in total chamber number did not exhibit a significant correlation with nest length (*p* > 0.05) (Figure 6b). Therefore, in this study, the number of chambers in *O. taurus* is primarily influenced by the internal diameter of the nest tube, whereas the chamber numbers for *M. spissula*, *A. septemspinosum*, and *M. sculpturalis* are more closely associated with nest tube length.

### 3.7. Pollen Spectrum of Bee Bread

**Figure 7:**
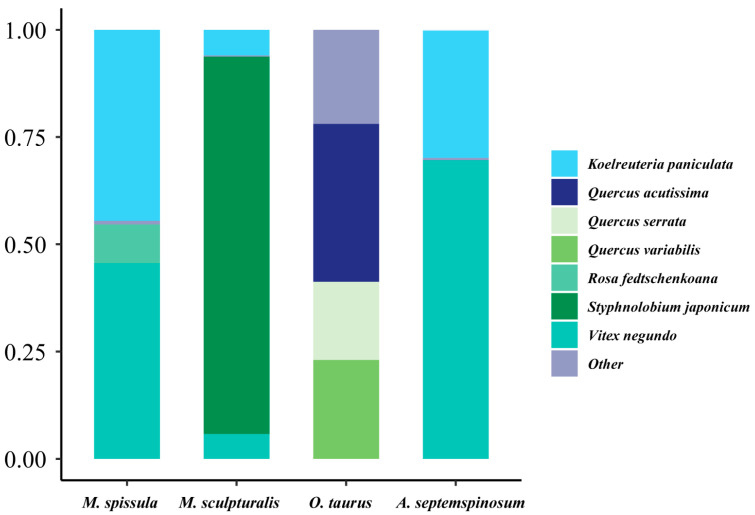
The percentage of pollen types of bee bread of the four species of leafcutting bees. Other plant species are presented in Supplementary Material Table S1.

High-throughput sequencing of the larval diet in four species of leafcutting bees revealed that the food sources originated from 48 plant species, belonging to 33 genera, 24 families, and 17 orders (Table S1). Among these, there are nine species from the Fagaceae family, five species each from the Rosaceae and Compositae families, and three species from the Fabaceae family. The dominant plant species for *M. spissula* were *Vitex negundo* and *Koelreuteria paniculata*, accounting for 45.56% and 44.54% of the pollen, respectively. For *M. sculpturalis*, the predominant plant species was *Styphnolobium japonicum* (87.98%), while *O. taurus* favored *Quercus acutissima* (36.84%). *Vitex negundo* also emerged as a dominant species for *M. sculpturalis* (69.62%) (Figure 7). Notably, 14 plant species (29.17%) were consumed by all nesting bee species, whereas 18 plant species (16.2%) were classified as special, interacting solely with one bee species. Both *Vitex negundo* and *Koelreuteria paniculata* exhibited relatively high abundance, with these two plants making significant contributions to the diets of *A. septemspinosum*, *M. spissula*, and *M. sculpturalis* at 99.31%, 90.03%, and 11.79%, respectively.

**Figure 8:**
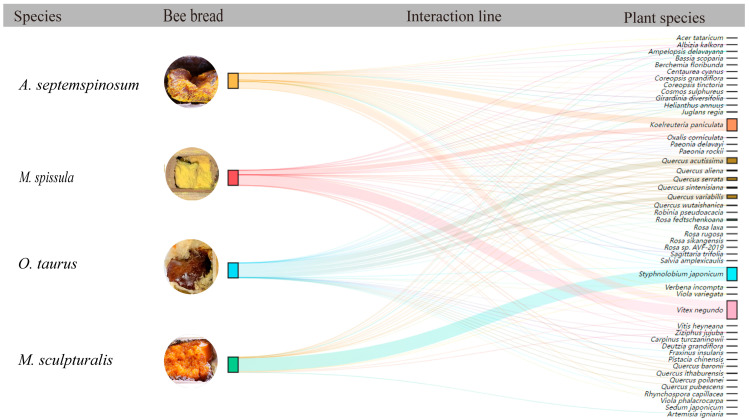
The interaction network relationship between pollen type of bee bread and leafcutting bees.

Through the analysis of the interactions among food sources within the nesting tubes of four species of solitary bees, the results indicate the establishment of 113 interaction networks between the four bee species and 48 plant species (Figure 8). *M. spissula* exhibited the highest interaction intensity, while *M. sculpturalis* displayed the lowest, suggesting that *M. spissula* pollinates the greatest number of plant species (36 species), whereas *M. sculpturalis* pollinates the fewest (19 species). Based on the degree of species specialization (d’), the low species specialization values of these four nesting bee species indicate that they mostly visit common plant species in their habitat. The asymmetry in interspecific interactions reveals that *M. spissula* interacts with the highest number of specialized plants, while *M. sculpturalis* interacts with the fewest, indicating that there are more specialized plants reliant on *M. spissula* for pollination and fewer specialized plants dependent on *M. sculpturalis* (Table 6).

## 4. Discussion

Long-term data indicate significant spatiotemporal fluctuations in the abundance, diversity, and activity patterns of solitary bees both between and within genera [41]. This study found that nesting activity peaks of four leafcutter bee species occur from late spring to late summer, consistent with Michener’s (2007) observation that summer is a period of particularly active nesting behavior for leafcutter bees [42]. During this period, their nesting strategy shifts from constructing temporary nest chambers for immediate egg-laying to building more stable seasonal burrows that provide long-term shelter for larval development. The abundant leaf resources in summer provide ample materials for nest construction; meanwhile, this period is also characterized by high species richness and diversity, aligning with other studies emphasizing summer as a critical period for honeybee foraging activities and plant–pollinator interactions [43]. Further analysis reveals significant temporal mismatches in the nesting peaks of the three leafcutter bee species studied: *O. taurus* peaks in June, *M. spissula* in July, and *M. sculpturalis* in August. In contrast, *A. septemspinosum* did not exhibit distinct nesting peaks in any month. This temporal mismatch may stem from the synchronization of nesting activities with the seasonal abundance of critical food resources such as pollen and nectar [44,45,46]. It may also be influenced by climatic factors such as temperature, rainfall, and the rhythmic activity of predators [47,48].

Climatic conditions influence the richness, composition, and morphology of plant communities and the nesting biology of bees, thereby indirectly affecting the reproduction of solitary bees [49]. In this experiment, Pearson correlation analysis revealed a positive correlation between the activity of leafcutter bees and rainfall and temperature. This finding contradicts studies by Kunjwal et al. (2016) and Marinho et al. (2017), indicating that foraging peaks of *Megachile* spp. occur in months with lower rainfall [41,50]. However, in agreement with Kaushik Pramanik, it is suggested that, during seasons with ample rainfall, plant growth is more vigorous, providing abundant nesting and food resources for bees [19]. Solitary bees, as typical poikilotherms, exhibit significantly increased metabolic rates and muscle activity efficiency within a certain temperature range (below their tolerance threshold) with elevated environmental temperatures, enhancing their activity in behaviors such as flight, foraging, and nesting [51]. Therefore, higher temperatures and rainfall in summer enhance bee activity, consistent with findings by Rehan and Richards (2010), who observed that these favorable seasonal climatic conditions and abundant floral resources promote bee reproduction and nesting behaviors [52].

Solitary bees exhibit significant differences in nesting behavior, phenology, and foraging preferences [53]. Within the scope of this study, the four solitary bee species examined displayed distinct choices in nesting materials. Generally, species that utilize leaves and mud for nesting (such as *M. spissula* and *O. taurus*) showed significantly higher nesting numbers compared to those that employ resin, pebbles, or cotton fluff (such as *M. sculpturalis* and *A. septemspinosum*). This disparity is likely linked to the energy costs associated with the collection of different materials [54]. For female solitary bees that seek and transport resin, pebbles, and cotton fluff, additional time and effort are required to locate suitable gathering sites and select appropriate material types (considering factors such as size and texture). Consequently, they must spend more time away from their nests and increase their flower visitation frequency to meet the elevated energy demands resulting from foraging. Within solitary bee communities, increased foraging duration and distance correspondingly lead to a reduction in nesting numbers [55]. As foraging flight time costs escalate, the resources allocated to offspring diminish [56,57], which subsequently lowers the overwintering larval survival rates of bees [58]. Furthermore, prolonged absences from the nest may heighten the risk of parasitism and nest invasion, further reducing offspring survival rates [59,60].

The diameter of nesting tubes plays a crucial role in attracting bees for nesting. In this study, 69% of leafcutter bees opted to nest in tubes with a diameter of 6–10 mm, which aligns with findings from [61,62]. We found that only *M. spissula* constructs nests in diameters ranging from 2–4 mm, as the average thorax width of female *M. spissula* is only 2.85 ± 0.22 mm, allowing them to nest in smaller burrows, while other species have thorax widths above 4–6 mm, with a minimum nest inner diameter of 4–6 mm. Therefore, the nest tube diameter of leafcutter bees is positively correlated with their thorax width. According to previous research, *M. sculpturalis* requires cavities with a minimum diameter of 8 mm, and its nests contain brood cells constructed individually for each offspring [63,64]. In our study, the average nest diameter of *M. sculpturalis* was approximately 8.86 mm, consistent with previous findings. Interestingly, around 10.26% of the nests had a diameter smaller than 8 mm, a phenomenon rarely observed in *M. sculpturalis* before. The body width of *M. spissula, O. taurus*, and *A. septemspinosum* is relatively small, yet their chosen range of burrow sizes is larger than that of the larger-bodied *M. sculpturalis*. This indicates that smaller-bodied species demonstrate greater adaptability to variations in burrow sizes [65]. Not all solitary Hymenoptera prefer small-diameter cavities, as excessively small cavity diameters restrict the oviposition rate of bees [66]. In situations where cavities are small, bees must locate more than one cavity for oviposition. Conversely, in cases of large cavity sizes, solitary female bees require additional nest-building materials to cover the cavities. Both phenomena incur higher energy costs for bees, ultimately leading to negative impacts on their reproductive efficiency [67].

In this study, the preferred nesting tube lengths for bees ranged from 131 to 170 mm, with some tubes reaching lengths of 180 to 230 mm, which aligns with the findings of [61]. Our research indicates that the number of nesting chambers increases with the length of the tubes, a trend that is particularly pronounced in *M. spissula*, *A. septemspinosum*, and *M. sculpturalis*, while *O. taurus* exhibited an increase that was not statistically significant. However, it is anticipated that the influence of nest tube length will diminish once the number of brood cells exceeds a critical threshold. Females construct a limited number of brood cells for male offspring at the entrance of the nest [68]. Gruber et al. (2011) noted a positive correlation between nest cavity length less than 150 mm and an increase in male offspring [69], attributed to the protection of economically valuable female offspring from parasitic effects [60]. Therefore, shorter nest tubes would constrain the remaining space for constructing brood cells for female offspring.

A total of 48 food sources were identified for four species of leafcutting bees. Our findings indicate that all four species of leafcutting bees are polylectic, collecting pollen from various plant families, including Fagaceae, Rosaceae, Compositae, and Fabaceae. Torretta and Durante (2011), dos Santos (2020), and others have found that many leafcutting bees exhibit a clear preference for the Rosaceae family [26,70]. R.H. Raina (2020) discovered a preference of leafcutting bees for plants in the Fabaceae family [18]. However, our study reveals a greater preference of leafcutting bees for plants in the Fagaceae family, with nine species, followed by five in the Rosaceae family, and only three in the Fabaceae family. This preference could be attributed to the higher abundance of Fagaceae family plants at the study site (such as *Quercus variabilis*, *Quercus acutissima*, *Quercus serrata*, *Quercus wutaishanica*, *Quercus aliena*), which may influence foraging activities of bees due to environmental factors. Although leafcutting bees visit a greater variety of plants from the Fagaceae and Rosaceae families, the pollen contribution is higher from *Vitex negundo* and *Koelreuteria paniculata*, especially in *A. septemspinosum* and *M. spissula*, accounting for over 90% of the total food intake. The flowering periods of frequently visited plants in this study (particularly from the Fagaceae and Fabaceae families) occur between May and July, aligning with the peak activity periods of these bees [71], indicating a synchronization between bee foraging periods and plant flowering periods to ensure an adequate food and nesting resource availability [72,73]. The four species of bees studied exhibit polylecty and cavity-nesting behaviors, positioning them as potential pollinators for various cultivated crops, and they may represent suitable candidates for future crop pollination management.

## 5. Conclusions

This study documented the seasonal activity patterns, nest structure characteristics, and foraging plants of four species of leafcutting bees through 1451 nesting traps. Nest-building activities of the leafcutting bees are primarily concentrated in the late spring and summer seasons, with temperature and rainfall being key influencing factors. Each species exhibits distinct characteristics in the materials used for constructing brood cells and nest partitions, as well as preferences for nest tunnel dimensions (length, diameter). A total of 48 plant species from 24 families, with 14 species representing shared pollen sources, were recorded as pollen sources visited by female bees. The findings of this study provide important insights for relevant conservation efforts.

## Figures and Tables

**Table 1:** The average and standard deviation of environmental parameters, as well as the Pearson regression coefficients and estimated *p*-values between total bee abundance and each environmental parameter (temperature, precipitation, and wind speed).

Years	Temperature (°C)				Precipitation (mm)				Wind Speed (km/h)			
	Average	sd	r	*p*-Value	Average	sd	r	*p*-Value	Average	sd	r	*p*-Value
2019	15.47	9.99	0.58	0.04	1.34	1.31	0.49	0.10	1.41	0.17	−0.20	0.51
2020	15.56	9.48	0.88	0.001	1.59	1.81	0.76	0.003	1.52	0.21	0.13	0.67
2021	15.66	9.38	0.88	0.001	2.56	3.33	0.68	0.01	1.68	0.18	−0.08	0.79
2022	15.96	10.28	0.82	0.001	1.57	2.06	0.45	0.13	1.54	0.25	0.15	0.62

**Table 2:** Materials for nest partitions and sealing materials in four species of leafcutting bees, as well as the timing of nesting activity.

Species	Partition	Sealing	Nesting Period
*M. spissula*	Leaves	Leaves	7–8 months
*M. sculpturalis*	Resin, mud	Resin, pebbles, mud	7–8 months
*O. taurus*	Mud	Mud	5–6 months
*A. septemspinosum*	Cotton fluff	Leaf mix	7–9 months

**Table 3:** The average thoracic width and nest inner diameter preferences of four species of leafcuting bees.

Species	Thorax Width (mm) ± SE (n = 10)		The Number of Nest-Building Inner Diameters (mm)					
	Female	Male	2–4 mm	4–6 mm	6–8 mm	8–10 mm	10–12 mm	12–14 mm
*M. spissula*	2.85 ± 0.22	2.49 ± 0.25	22	262	387	59	2	
*M. sculpturalis*	6.81 ± 0.49	5.62 ± 0.73			8	45	24	
*O. taurus*	5.10 ± 0.66	3.32 ± 0.36		53	201	219	54	4
*A. septemspinosum*	4.35 ± 0.34	4.8 ± 0.53		2	42	51	19	1

**Table 4:** The average body length and nest-building length preferences of four species of leafcutting bees.

Species	Body Length (mm) ± SE (n = 10)		The Quantity of Nest-Building Lengths (mm)					
	Female	Male	10–50 mm	51–90 mm	91–130 mm	131–170 mm	171–210 mm	211–250 mm
*M. spissula*	9.70 ± 0.73	8.30 ± 0.74	72	155	209	194	79	19
*M. sculpturalis*	23.02 ± 1.93	17.22 ± 1.92	4	9	25	28	12	0
*O. taurus*	11.96 ± 1.03	9.63 ± 1.64	45	91	131	172	87	3
*A. septemspinosum*	13.29 ± 0.94	14.06 ± 1.79	5	21	34	40	12	3

**Table 5:** The range of nest cell numbers constructed by four species of leafcutting bees, the range of brood cell numbers, as well as the mean nest cell number (±SE) and mean brood cell number (±SE).

Species	Sample Size (n)	Total Chambers		Nursery Chambers	
		Range	Mean ± SE	Range	Mean ± SE
*M. spissula*	728	1–22	5.82 (0.11)	1–15	4.50 (0.09)
*M. sculpturalis*	78	1–9	3.53 (0.18)	1–9	2.62 (0.18)
*O. taurus*	529	1–27	9.88 (0.23)	1–27	8.93 (0.22)
*A. septemspinosum*	116	1–12	5.53 (0.25)	1–12	5.32 (0.0.25)

**Table 6:** Parameters of the interaction network relationship between pollen type of bee bread and the four species of leafcutting bees.

Species	Number of Interacting Species	Species Strength	Specialization (d’)	Interspecific Asymmetry
*M. spissula*	36	17.91	0.06	0.46
*M. sculpturalis*	19	5.75	0.04	0.25
*O. taurus*	31	14.75	0.14	0.44
*A. septemspinosum*	27	9.58	0.03	0.31

## Data Availability

The original contributions presented in this study are included in the article. Further inquiries can be directed to the corresponding author.

## Supplementary Materials

The following supporting information can be downloaded at: https://www.mdpi.com/article/10.3390/insects16080831/s1, Table S1: Pollen spectrum of bee bread.
