# Supplementary material for: Nesting and Foraging Preferences of Four Sympatric Species of Cavity-Nesting Leafcutting Bees (Hymenoptera: Megachilidae)
Source: Insects. 2025 Aug 11;16(8):831. doi: 10.3390/insects16080831 (PMC12386985; doi:10.3390/insects16080831)
Supplement: Supplementary file 1 [file insects-16-00831-s001.zip › insects-3734127-supplementary.pdf]

Table S1. Pollen spectrum of bee bread.

Order	Family	Genus	Species
Lamiales	Verbenaceae	*Verbena*	*Verbenaincompta*
	Lamiaceae	*Vitex*	*Vitexnegundo*
Poales	Cyperaceae	*Rhynchospora*	*Rhynchosporacapillacea*
Juglandales	Juglandaceae	*Juglans*	*Juglansregia*
Saxifragales	Paeoniaceae	*Paeonia*	*Paeoniadelavayi*
			Paeoniarockii
	Crassulaceae	*Sedum*	*Sedumjaponicum*
Malpighiales	Violaceae	*Viola*	*Violavariegata*
			Violaphalacrocarpa
Asterales	Compositae	*Centaurea*	*Centaureacyanus*
		Helianthus	*Helianthusannuus*
		Cosmos	*Cosmossulphureus*
		Coreopsis	*Coreopsisgrandiflora*
			Coreopsistinctoria
		Artemisia	*Artemisiaigniaria*
Fagales	Fagaceae	*Quercus*	*Quercusacutissima*
			Quercusserrata
			Quercusvariabilis
			Quercuswutaishanica
			Quercusaliena
			Quercusbaronii
			Quercusithaburensis
			Quercuspubescens
			Quercussintenisiana
		Cyclobalanopsis	*Quercuspoilanei*
Magnoliales	Annonaceae	*Uvaria*	*Salviaamplexicaulis*
Oleales	Oleaceae	*Fraxinus*	*Fraxinusinsularis*
Vitales	Vitaceae	*Ampelopsis*	*Ampelopsisdelavayana*
		Vitis	*Vitisheyneana*
Rosales	Fabaceae	*Albizia*	*Albiziakalkora*
		Robinia	*Robiniapseudoacacia*
		Styphnolobium	*Styphnolobiumjaponicum*
		Deutzia	*Deutziagrandiflora*
	Rosaceae	*Rosa*	*Rosafedtschenkoana*
			Rosalaxa
			Rosarugosa
			Rosasikangensis

			Rosasp.AVF-2019
Fagales	Betulaceae	*Carpinus*	*Carpinusturczaninowii*
Caryophyllales	Polygonaceae	*Girardinia*	*Girardiniadiversifolia*
	Amaranthaceae	*Bassia*	*Bassiascoparia*
Rhamnales	Rhamnaceae	*Ziziphus*	*Ziziphusjujuba*
		Berchemia	*Berchemiafloribunda*
Sapindales	Sapindaceae	*Koelreuteria*	*Koelreuteriapaniculata*
	Aceraceae	*Acer*	*Acertataricum*
	Anacardiaceae	*Pistacia*	*Pistaciachinensis*
Alismatales	Alismataceae	*Sagittaria*	*Sagittariatrifolia*
Oxalidales	Oxalidaceae	*Oxalis*	*Oxaliscorniculata*
